# Sub-growth-inhibitory concentrations of omadacycline inhibit *Staphylococcus aureus* haemolytic activity *in vitro*

**DOI:** 10.1093/jacamr/dlab190

**Published:** 2021-12-22

**Authors:** Alisa W Serio, S Ken Tanaka, Kelly Wright, Lynne Garrity-Ryan

**Affiliations:** Paratek Pharmaceuticals, Inc., 1000 First Ave, Suite 200, King of Prussia, PA 19406, USA

## Abstract

**Objectives:**

To evaluate the effect of sub-growth-inhibitory concentrations of omadacycline on *Staphylococcus aureus* ATCC 10832 haemolytic activity *in vitro*.

**Methods:**

Following determination of the MICs of omadacycline and comparator antibiotics, the strain was grown in the presence of individual antibiotics and the percentage of haemolysis assayed; ‘washout’ experiments were performed with omadacycline only.

**Results:**

Omadacycline inhibited *S. aureus* haemolytic activity *in vitro* at sub-growth-inhibitory concentrations. Inhibition was maintained at least 4 h after removal of extracellular drug.

**Conclusions:**

Omadacycline’s *in vitro* potency and suppression of virulence factors might contribute to its efficacy in the treatment of acute bacterial skin and skin structure infections and community-acquired bacterial pneumonia caused by virulent strains of *S. aureus*. This finding could be relevant for other organisms and virulence factors that depend on new protein synthesis.

## Introduction

Certain bacteria produce virulence factors, which contribute to the ability of a microorganism to cause an infection, and the severity of an infection—e.g. causing tissue destruction by interfering with the host response to infection, or causing uncontrolled inflammation.^1–3^ Favourable treatment outcomes can be achieved by killing the infecting organism or by preventing its multiplication, and agents that can also prevent or inhibit virulence factor production and/or activity may offer additional benefit. In animal models of *Staphylococcus aureus* infection, α-haemolysin, a pore-forming exotoxin that can damage the host cell’s plasma membrane, has been shown to be a key virulence factor.^1–4^

Virulence factor production largely depends on protein synthesis, and protein synthesis inhibitor antibiotics have been shown to suppress the production of proteinaceous exotoxins in organisms such as *S. aureus.*^5^ Indeed, the IDSA clinical practice guidelines for the treatment of MRSA infections state that protein synthesis inhibitor antibiotics such as clindamycin and linezolid may be considered as adjunctive therapy in some scenarios.^6^ Exposure to protein synthesis inhibitors at levels at or below concentrations required to inhibit bacterial multiplication may decrease α-haemolysin expression^7–9^ and could therefore provide continued antivirulence effects independent of the agent’s effect on organism growth inhibition. Therefore, the use of recommended doses of protein synthesis inhibitors to treat infections caused by *S. aureus* could provide the necessary exposure to inhibit *S. aureus* multiplication, with the possible added benefit of continued inhibition of the production of *S. aureus*-associated virulence factors.

Omadacycline, a novel aminomethylcycline antibiotic derived from the tetracycline class of bacterial protein biosynthesis inhibitors, shows activity against methicillin-susceptible *S. aureus* (MSSA) and MRSA.^10^ It is approved in the United States for the treatment of adult patients with community-acquired bacterial pneumonia and acute bacterial skin and skin structure infections caused by susceptible microorganisms, including *S. aureus.*^10^ We sought to determine the durability of inhibition and effect of sub-growth-inhibitory concentrations of omadacycline on *S. aureus* haemolytic activity.

## Materials and methods

All experiments were conducted with MSSA Wood 46 (ATCC 10832), which secretes high levels of α-haemolysin.^11^*S. aureus* cultures were grown in cation-adjusted Mueller Hinton II (MH) broth media (Becton Dickinson) and on tryptic soy agar plates with 5% sheep blood (Northeast Laboratory Services) at 37°C. MICs of omadacycline and comparator antibiotics were determined using CLSI methodology in cation-adjusted MH broth.^12^ Comparators included tetracycline, clindamycin, linezolid, vancomycin, and cephalothin.

### Growth of S. aureus with antibiotics

Antibiotics were added to broth cultures during mid-log growth. At 4 h after drug addition, cfu/mL were determined. Filtered supernatants were tested for haemolytic activity by incubation with rabbit erythrocytes. Control samples included drugs with media and no bacteria, to show that the drug alone does not cause haemolysis, and drugs added to fully grown cultures before filter sterilizing, to show that the drug was not interfering with detection of haemolytic activity.

In washout experiments, omadacycline was added to mid-log broth cultures for 1 h. Spent media, after centrifugation, was removed, and pelleted bacteria were resuspended in the same volume of fresh media without drug and incubated for another 4 h.

### Haemolysis assay

Filtered culture supernatant aliquots were diluted and mixed with an equal volume of 2% defibrinated rabbit blood (Remel) in PBS in microtitre plates. MH broth was used as a control for 0% haemolysis; MH broth containing lysis solution [9% (v/v) solution of Triton-X-100 (Promega)] was used as a control for 100% haemolysis. Purified *S. aureus* α-haemolysin (Toxin Tech, HP101) was also included as a control (a concentration curve was constructed for each plate: 100 μL of 1 mg/mL stock reagent was diluted in 900 μL of MH broth with 1% DMSO; 500 μL was then diluted with 500 μL of MH broth with 1% DMSO, and this was repeated for a total of seven dilutions). After incubation at 37°C for 1 h, the microtitre plates were centrifuged and the supernatant transferred to another microtitre plate for analysis. The average absorbance at 450 nm (A_450 nm_) of media-only wells was subtracted from the A_450 nm_ of all other wells as background, recorded via a Victor V plate reader. Data were expressed as: % haemolysis = (sample A_450 nm_/average lysed A_450 nm_) × 100. The percentage haemolysis data for each dilution series were plotted with Excel, and XLfit was used to determine the volume of sample required to cause 50% haemolysis, which equals 1 haemolytic unit. Haemolytic units/mL culture were calculated and dilution plating results were used to convert data to haemolytic units/cfu.

### Data availability

Paratek Pharmaceuticals has a commitment to ensure that data are available to regulators and researchers when permitted, feasible and appropriate. Requests for data may be submitted to medinfo@paratekpharma.com.

## Results

**Figure 1.**
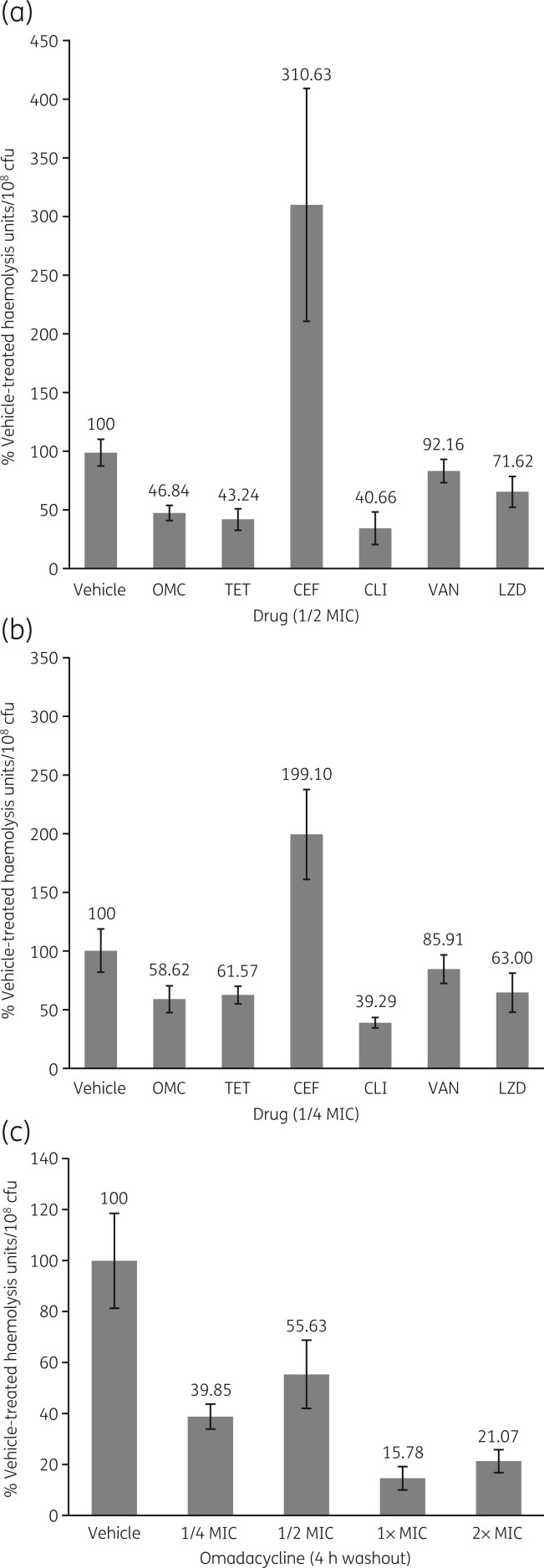
Haemolytic activity of *S. aureus* Wood 46 after 4 h growth with 1/2 MIC (a) and 1/4 MIC (b) of omadacycline (OMC), tetracycline (TET), cephalothin (CEF), clindamycin (CLI), vancomycin (VAN), or linezolid (LZD). Vehicle = 0.0003% DMSO in Mueller Hinton II (MH) broth. Data represent the mean of five cultures; error bars indicate standard deviations. Panel (c) shows haemolytic activity of *S. aureus* Wood 46 grown for 1 h with the indicated concentration of omadacycline, followed by 4 h growth without drug. Vehicle = 0.0025% DMSO in MH broth. Data represent the mean of three cultures; error bars indicate standard deviations. Values <100% indicate more inhibition of haemolytic activity than the vehicle control.

*S. aureus* cultures treated with 1/2 or 1/4 the MIC of omadacycline for 4 h showed haemolysis units/10^8^ cfu of 47% and 59% of vehicle-treated cultures, respectively (Figure 1a and b). These values were comparable to those seen with 1/2 or 1/4 the MIC of comparator protein synthesis inhibitors: tetracycline (43% and 62%), clindamycin (41% and 40%) and linezolid (72% and 63%); MICs for respective antibiotics are listed in Table 1. In contrast, when cultures were treated with sub-growth inhibitory concentrations of cell wall inhibitor antibiotics, there was less inhibition of haemolysis with vancomycin (92% and 86% of vehicle-treated haemolysis units/cfu, with 1/2 MIC and 1/4 MIC, respectively), and there was even an increase in haemolysis with cephalothin (311% and 199% of vehicle-treated haemolysis units/cfu, with 1/2 MIC and 1/4 MIC, respectively). In washout experiments, exposure to as little as 1/4 of the MIC of omadacycline for 1 h decreased the haemolysis units/10^8^ cfu by 60% for 4 h following removal of the drug (Figure 1c).

**Table 1.** MICs (mg/L) of omadacycline and comparator agents evaluated against *S. aureus* Wood 46

Antibiotic	MIC	1/2 MIC	1/4 MIC	1/8 MIC
Omadacycline	0.25	0.12	0.06	0.03
Tetracycline	0.12	0.06	0.03	0.015
Cephalothin	0.25	0.12	0.06	0.03
Clindamycin	0.06	0.03	0.015	0.008
Vancomycin	1	0.5	0.25	0.12
Linezolid	1	0.5	0.25	0.12

## Discussion

Omadacycline inhibited *S. aureus* haemolytic activity *in vitro* at sub-growth-inhibitory concentrations, and inhibition was maintained for at least 4 h after removal of extracellular drug. This is the first study to demonstrate these effects with omadacycline and, to our knowledge, comparable data on the inhibition of haemolytic activity in this strain of *S. aureus* are not available for other tetracyclines. The effect of sub-growth-inhibitory concentrations of antibiotics on *S. aureus* virulence factor expression has been found to vary between drugs and among virulence factors.^13^ Previously, an antitoxin effect for *S. aureus* has been largely associated with clindamycin and more recently with linezolid, with limited information available on tetracyclines.^7^^,^^14–16^ However, most currently available protein synthesis inhibitors are less desirable monotherapy options for *S. aureus* infections due to resistance in *S. aureus* to clindamycin and azithromycin,^17^ safety concerns with other agents, including cardiac issues with azithromycin,^18^*Clostridioides difficile* colitis associated with clindamycin,^19^ and myelosuppression caused by linezolid.^20^ In future studies, it would be of interest to explore the effects of newer anti-ribosomal agents, such as tedizolid, in terms of suppression of haemolytic activity. Additionally, because it is not known how the suppression of haemolytic activity observed with omadacycline compares with that seen with other tetracyclines, such as doxycycline and minocycline, investigating this in future studies would also be worthwhile. An important limitation of the current study is that it examined haemolytic activity in a single strain of *S. aureus*, which is known to be a high-level producer of alpha toxin.^21^ The clinical relevance of the findings for *S. aureus* infections could be further investigated using strains of MRSA such as ST22, ST59, ST239, USA300 MRSA, or USA100. Additionally, analyses for other virulence factors, including those in group A streptococci, are warranted given the burden of toxin production for these infection types.

### Conclusions

The suppression of virulence factors, in addition to the *in vitro* potency of omadacycline, may contribute to the efficacy of omadacycline for community-acquired bacterial pneumonia and acute bacterial skin and skin structure infections caused by virulent strains of *S. aureus*. This finding could apply to other organisms and other virulence factors that require new protein synthesis to establish and enable the progression of disease.

## References

[1] Bramley AJ , Patel AH, O’Reilly M et al Roles of alpha-toxin and β-toxin in virulence of *Staphylococcus aureus* for the mouse mammary gland. Infect Immun 1989; 57: 2489–94. 2744856 10.1128/iai.57.8.2489-2494.1989 PMC313475

[2] O’Callaghan RJ , Callegan MC, Moreau JM et al Specific roles of alpha-toxin and β-toxin during *Staphylococcus aureus* corneal infection. Infect Immun 1997; 65: 1571–8. 9125532 10.1128/iai.65.5.1571-1578.1997 PMC175175

[3] Patel AH , Nowlan P, Weavers ED et al Virulence of protein A-deficient and alpha-toxin-deficient mutants of *Staphylococcus aureus* isolated by allele replacement. Infect Immun 1987; 55: 3103–10. 3679545 10.1128/iai.55.12.3103-3110.1987 PMC260034

[4] Vandenesch F , Lina G, Henry T. *Staphylococcus aureus* hemolysins, bi-component leukocidins, and cytolytic peptides: a redundant arsenal of membrane-damaging virulence factors? Front Cell Infect Microbiol 2012; 2: 12. 22919604 10.3389/fcimb.2012.00012 PMC3417661

[5] Campbell AJ , Dotel R, Blyth CC et al Adjunctive protein synthesis inhibitor antibiotics for toxin suppression in *Staphylococcus aureus* infections: a systematic appraisal. J Antimicrob Chemother 2019; 74: 1–5. 30307507 10.1093/jac/dky387

[6] Liu C , Bayer A, Cosgrove SE et al Clinical Practice Guidelines by the Infectious Diseases Society of America for the treatment of methicillin-resistant *Staphylococcus aureus* infections in adults and children: Executive Summary. Clin Infect Dis 2011; 52: 285–92. 21217178 10.1093/cid/cir034

[7] Ohlsen K , Ziebuhr W, Koller KP et al Effects of subinhibitory concentrations of antibiotics on alpha-toxin (hla) gene expression of methicillin-sensitive and methicillin-resistant *Staphylococcus aureus* isolates. Antimicrob Agents Chemother 1998; 42: 2817–23. 9797209 10.1128/aac.42.11.2817 PMC105949

[8] Bernardo K , Pakulat N, Fleer S et al Subinhibitory concentrations of linezolid reduce *Staphylococcus aureus* virulence factor expression. Antimicrob Agents Chemother 2004; 48: 546–55. 14742208 10.1128/AAC.48.2.546-555.2004 PMC321544

[9] Doss SA , Tillotson GS, Amyes SG. Effect of sub-inhibitory concentrations of antibiotics on the virulence of *Staphylococcus aureus*. J Appl Bacteriol 1993; 75: 123–8. 8407672 10.1111/j.1365-2672.1993.tb02756.x

[10] Paratek Pharmaceuticals, Inc. NUZYRA (omadacycline) prescribing information. Revised October 2020. https://www.nuzyra.com/nuzyra-pi.pdf.

[11] Gray GS , Kehoe M. Primary sequence of the alpha-toxin gene from *Staphylococcus aureus* wood 46. Infect Immun 1984; 46: 615–8. 6500704 10.1128/iai.46.2.615-618.1984 PMC261580

[12] CLSI. *Methods for Dilution Antimicrobial Susceptibility Tests for Bacteria That Grow Aerobically. Approved Standard—Ninth Edition: M07*. 2012.

[13] Otto MP , Martin E, Badiou C et al Effects of subinhibitory concentrations of antibiotics on virulence factor expression by community-acquired methicillin-resistant *Staphylococcus aureus*. J Antimicrob Chemother 2013; 68: 1524–32. 23508621 10.1093/jac/dkt073

[14] Stevens DL , Ma Y, Salmi DB et al Impact of antibiotics on expression of virulence-associated exotoxin genes in methicillin-sensitive and methicillin-resistant *Staphylococcus aureus*. J Infect Dis 2007; 195: 202–11. 17191165 10.1086/510396

[15] Diep BA , Afasizheva A, Le HN et al Effects of linezolid on suppressing in vivo production of staphylococcal toxins and improving survival outcomes in a rabbit model of methicillin-resistant *Staphylococcus aureus* necrotizing pneumonia. J Infect Dis 2013; 208: 75–82. 23532096 10.1093/infdis/jit129 PMC3666136

[16] Hodille E , Rose W, Diep BA et al The role of antibiotics in modulating virulence in *Staphylococcus aureus*. Clin Microbiol Rev 2017; 30: 887–917. 28724662 10.1128/CMR.00120-16 PMC5608880

[17] Leclercq R. Mechanisms of resistance to macrolides and lincosamides: nature of the resistance elements and their clinical implications. Clin Infect Dis 2002; 34: 482–92. 11797175 10.1086/324626

[18] Zaroff JG , Cheetham TC, Palmetto N et al Association of azithromycin use with cardiovascular mortality. JAMA Netw Open 2020; 3: e208199. 32585019 10.1001/jamanetworkopen.2020.8199 PMC7301226

[19] Buffie CG , Jarchum I, Equinda M et al Profound alterations of intestinal microbiota following a single dose of clindamycin results in sustained susceptibility to *Clostridium difficile*-induced colitis. Infect Immun 2012; 80: 62–73. 22006564 10.1128/IAI.05496-11 PMC3255689

[20] Gerson SL , Kaplan SL, Bruss JB et al Hematologic effects of linezolid: summary of clinical experience. Antimicrob Agents Chemother 2002; 46: 2723–6. 12121967 10.1128/AAC.46.8.2723-2726.2002 PMC127358

[21] Leng BF , Qiu JZ, Dai XH et al Allicin reduces the production of α-toxin by *Staphylococcus aureus*. Molecules 2011; 16: 7958–68. 21921868 10.3390/molecules16097958 PMC6264299

[22] Serio AW , Tanaka SK, Wright K et al Subinhibitory concentrations of omadacycline inhibit *Staphylococcus aureus* hemolytic activity in vitro. Abstract 1202. Open Forum Infect Dis 2020; 7 Suppl 1: S622–3.

